# Cardioprotective Effect of a Virgin Olive Oil Enriched with Bioactive Compounds in Spontaneously Hypertensive Rats

**DOI:** 10.3390/nu11081728

**Published:** 2019-07-26

**Authors:** Alejandra Vazquez, Estefania Sanchez-Rodriguez, Félix Vargas, Sebastián Montoro-Molina, Miguel Romero, Juan Antonio Espejo-Calvo, Pedro Vilchez, Sara Jaramillo, Lucía Olmo-García, Alegría Carrasco-Pancorbo, Rafael de la Torre, Montserrat Fito, María-Isabel Covas, Emilio Martínez de Victoria, Maria Dolores Mesa

**Affiliations:** 1 Department of Biochemistry and Molecular Biology II, Institute of Nutrition and Food Technology “José Mataix”, Biomedical Research Center, University of Granada, Parque Tecnológico de la Salud, Avenida del Conocimiento s/n, 18100 Armilla, Granada, Spain; 2 Department of Physiology, Phaculty of Medicine, University of Granada, Parque Tecnológico de la Salud, Avenida del Conocimiento s/n, 18100 Armilla, Granada, Spain; 3 Department of Pharmacology, Phaculty of Pharmacy, University of Granada, Campus Cartuja s/n, 180710 Armilla, Granada, Spain; 4 Instituto para la Calidad y Seguridad Alimentaria S.L. (ICSA)-TECNOFOOD I+D SOLUCIONES S.L., Avenida de la Hispanidad 17, 18320 Santa Fe, Granada, Spain; 5 Laboratorio CM Europa S.L., Polígono Industrial “Cañada de la Fuente”, Carretera Fuensanta, s/n, 23600 Martos, Jaén, Spain; 6 Vegetable By-Products of Mediterráneo, SL, Cl Isla Menor CEP Jose Maria Blanco SN, 41010 Seville, Spain; 7 Fat Institute: Department of Food Phytochemistry Campus of the Pablo de Olavide University, Building 46 Ctra. De Utrera, km. 1, 41013 Seville, Spain; 8 Department of Analytical Chemistry, Faculty of Science, University of Granada, Ave. Fuentenueva s/n, 18071 Granada, Spain; 9 Cardiovascular Risk and Nutrition Research Group, Hospital del Mar Medical Research Institute (IMIM), Dr. Aiguader 88, 08003 Barcelona, Spain; 10 Spanish Biomedical Research Networking Centre, Physiopathology of Obesity and Nutrition (CIBEROBN), Instituto de Salud Carlos III. Monforte de Lemos 3-5, 28029 Madrid, Spain; 11 NUPROAS Handelsbolag, Nackã, Sweden, NUPROAS HB, Spanish Office: Apartado de Correos 93, 17242 Girona, Spain; 12 Department of Physiology, Institute of Nutrition and Food Technology “José Mataix”, Biomedical Research Center, University of Granada, Health Technology Park, Avd of Conocimiento s/n, 18100 Armilla, Granada, Spain; 13 Biosanitary Research Institute of Granada, 18014 Granada, Spain

**Keywords:** extra virgin olive oil, phenolic compounds, hypertension, endothelial function

## Abstract

Olive oil and its derivatives have been described to exert beneficial effects on hypertensive states and cardiovascular disease prevention. We studied the effects of chronic consumption of extra virgin olive oil (EVOO), enriched in bioactive compounds from olive fruit and leaves, on blood pressure, endothelial function, oxidative and inflammatory status, and circulating cholesterol levels, in spontaneously hypertensive rats (SHR). Thirty SHR were randomly assigned to three groups: a control untreated SHR group, an SHR group (1 mL/rat/day) of a control olive oil (17.6 mg/kg of phenolic compounds), and an SHR group (1 mL/rat/day) of the enriched EVOO (750 mg/kg of phenolic compounds) for eight weeks. Ten Wistar Kyoto rats (WKY) were included as healthy controls. Long-term administration of the enriched EVOO decreased systolic blood pressure and cardiac hypertrophy, and improved the ex vivo aortic endothelial dysfunction measured in SHR. Moreover, enriched oil supplementation reduced the plasma levels of Angiotensin II and total cholesterol, and the urinary levels of endothelin-1 and oxidative stress biomarkers, while pro-inflammatory cytokines were unaffected. In conclusion, sustained treatment with EVOO, enriched in bioactive compounds from the olive fruit and leaves, may be an effective tool for reducing blood pressure and cholesterol levels alone or in combination with pharmacological anti-hypertensive treatment.

## 1. Introduction

Hypertension is one of the most powerful risk factors for cardiovascular events, including myocardial infarction and stroke, and its effective treatment reduces cardiovascular morbidity and mortality [1]. However, the efficacy of anti-hypertensive drugs is still not entirely satisfactory, and new approaches should be considered [1]. Risks of adverse reaction and medication costs have focused attention on the use of alternative and complementary natural medicines. Among nutritional strategies, extra virgin olive oil (EVOO) has been proposed as a good cardioprotective tool. A systematic review and meta-analysis have evaluated the effect of high versus low polyphenol olive oil on cardiovascular disease risk factors in clinical trials. They found some evidence of the improvements in inflammatory markers and blood pressure, mainly high polyphenol oils conferring some cardiovascular benefits [2]. Yuvero et al. [3] reviewed findings about the effects of EVOO on cardiovascular risk factors, and the underlying mechanisms, encouraging future research in order to ascertain not only the effect, but also the real components responsible for the cardiovascular benefits. 

*Olea europaea* L. leaves have been used to combat high blood pressure, atherosclerosis, and diabetes [4]. The anti-hypertensive and cholesterol-lowering effects of these leaves are observed in experimental and clinical studies [5,6,7]. A reduction of blood pressure has been observed in spontaneously hypertensive rats (SHR) fed a diet enriched with EVOO during 12 weeks compared with a standard diet [8]. Sustained administration of oleanolic acid-enriched pomace olive oil in SHR improved the endothelial function conductance [9] and resistance arteries [10], by increasing endothelial nitric oxide synthase (eNOS) protein expression. Minor compounds from olive oil and olive leaves, such as oleuropein, have been recognized as responsible for acute endothelium-independent vasodilatory effects in isolated SHR aortae [11]. Moreover, it has been reported that sustained intake of an oleuropein-enriched olive leaf extract exerts anti-hypertensive effects on genetic hypertension by improving vascular function and reducing oxidative and inflammatory status in SHR [12]. These effects are associated with the inhibition of the angiotensin converting enzyme (ACE). Therefore, olive oil minor compounds and olive derivatives may be responsible for the anti-hypertensive effects, and the enrichment of VOOs with these compounds may improve its protective properties. 

The present study primarily evaluated the anti-hypertensive effect of an EVOO enriched in compounds obtained from olive fruits and leaves compared with a control olive oil in genetically hypertensive SHR. We also investigated the mechanisms involved in their anti-hypertensive effect, by evaluating vascular function ex vivo, as well as the antioxidant and anti-inflammatory status.

## 2. Materials and Methods 

### 2.1. Experimental Oils 

Olive oils were specially prepared for the study at the Cooperativa de San Francisco de Asís de Montefrío, Granada, Spain. An EVOO with high amounts of phenolic compounds was selected. Part of the EVOO was enriched selectively with three different extracts obtained from the olive oil and olive leaves, which contained mainly 200 mg/kg of hydroxytyrosol, 3,4-dihydroxyphenylglycol, and oleuropein, with a total amount of 750 mg/kg of phenolic compounds in the functional olive oil (FOO). Part of the selected EVOO was washed with alimentary ethanol to reduce the content of phenolic compounds to 17.6 mg/kg control oil (CO). Thus, the two experimental oils only differed in the content of phenolic compounds. Table 1 describes chemical characteristics of the experimental oils used in the study. Quantification of the individual phenolic compounds belonging to each chemical family is shown in Supplementary Table S1 [13]. The oils were prepared and frozen in daily aliquots in order to avoid oxidation. 

### 2.2. Experimental Design 

Animals were purchased from Janvier Labs, CEDEX, France. Thirty SHR of eight weeks of age were divided into three experimental groups (*n* = 10). One group was daily supplemented with 1 mL of the experimental functional olive oil (FOO) that is the EVOO enriched in bioactive compounds (SHR-FOO), the second group of hypertensive rats was daily supplemented with a 1 mL of the control oil (CO) (SHR-CO), and a third group of hypertensive rats (SHR) was used as the control and received 1 mL of water daily. In addition, 10 Wistar Kyoto healthy (WKY-H) male rats with the same age were included as normotensive healthy controls and received 1 mL of water daily. Oils and water were administered by using a rigid orogastric tube that went from the mouth to the stomach directly.

All rats had *ad libitum* access to food and water. Animals were fed on a standard maintenance diet (Panlab), with 76.2% carbohydrates (fiber 3.9%), 3.1% lipids, 16.1% protein, and 4.6 mineral-ashs. The animals were treated for 8 weeks with the enriched, functional, or control olive oils (SHR-FOO or SHR-CO groups, respectively), or with water in both SHR and WKY-H groups. The experiment was performed in accordance with the guidelines set by the European Community Council Directives for the ethical care of animals (86/609/EEC) and were approved by the ethical committee of Laboratory Animals of the University of Granada (Spain, permit number 18/07/2017/099).

The systolic blood pressure (SBP) and body weight (BW) were measured weekly during the course of the experiment. At the beginning of the study, every two weeks, and at the end of the 8 weeks of intervention, rats were introduced in metabolic cages (Panlab, Barcelon, Spain). Food and water intake were monitored and urine was collected for 24 h.

The administration of the oils was stopped 24 h before the end of the experiment in order to study the long-term effects of the active phenolics without the involvement of the effects of acute administration. After the 8 weeks of intervention, fasting rats were anesthetized with 2.5 mL per kg equitensin (i.p.), and drawn by abdominal aortic puncture by a beveled, which permits a fractional extraction, to determine plasma variables. To start, 1 mL of blood was drawn for the measurement of the plasma levels of Angiotensin II. This procedure permits an accurate measurement of this peptide, without the activation of the renin-angiotensin system due to the hemorrhage. The rest of the blood was used to measure the other plasma variables, which are not affected by hemorrhaging. The blood was centrifuged at 1750*× g* for 10 min at 4 °C. Aliquots of plasma were frozen immediately at −80 °C until analysis. Lastly, the rats were sacrificed by exsanguinations, and the kidneys, heart, and thoracic aorta were removed and weighed. The heart was divided into the right ventricle and the left ventricle plus septum. The length of the tibia was measured to normalize the morphological variables. 

### 2.3. Blood Pressure Determination

Tail SBP was measured weekly by tail-cuff plethysmography in conscious rats (LE 5001-Pressure Meter, Letica SA, Barcelona, Spain). At least seven determinations were made at every session, and the mean of the lowest three values within a range of 5 mmHg was the final SBP value.

### 2.4. Plasma Biochemical Analysis

Plasma electrolytes (sodium and potassium), low density lipoprotein (LDL) cholesterol, high density lipoprotein (HDL) cholesterol, total cholesterol, urea, and creatinine were measured by the autoanalyzer (Hitachi-912, Roche, Granada, Spain). Plasma angiotensin II (CEA 005Ra), urinary endothelin-1 (CEA482Ra), plasma interleukin 6 (IL-6, SEA079Ra), the tumor necrosis factor alpha (TNF-α; SEA133Mi), and the vascular endothelial growth factor (VEGF, SEA143Ra) were measured by the enzyme-linked immunosorbent assay (ELISA) kits referred above to Cloud Clone Corp, Katy, TX, USA. 

### 2.5. Urine Biochemical Analysis

At the end of the experimental period, the 24-h urine volume was measured, electrolytes (sodium and potassium), urea, and creatinine were measured by the autoanalyzer (Hitachi-912, Roche, Granada, Spain), and proteinuria by using the DC Protein Assay Kit (Bio-Rad, Madrid, Spain). Creatinine clearance, and the water and sodium balance were calculated in 24 h-urines as biomarkers of renal function. Total nitrate/nitrite in urine was measured by a colorimetric kit (Cayman Chemicals Company, Ann Arbor, MI, USA).

ELISA kits were used to measure 24 h urine oxidative stress biomarkers, F_2_-isoprostanes, and 8-hidroxy-2′-deoxyguanosine (8-OH-dG) (EA85, from Oxford Biomedical Research, Rochester Hills, MI, USA, and JAI-KOG-200SE, from Bio-Connect B.V. The Netherlands, respectively), and endothelin-1 (CEA482RA, from Cloud Clone Corp, Katy, TX, USA). 

### 2.6. Vascular Reactivity in Aortic Rings

Segments of thoracic aortic rings were mounted in an organ chamber filled with a Krebs solution (NaCl 118 mM, KCl 4.75 mM, NaHCO_3_ 25 mM, MgSO_4_ 1.2 mM, CaCl_2_ 2 mM, KH_2_PO_4_ 1.2 mM, and glucose 11 mM) at 37 °C, gassed with 95% O_2_ and 5% CO_2,_ and maintained at a resting tension of 2 g. Isometric tension was determined using an isometric force-displacement transducer (Letigraph 2000, Madrid, Spain) connected to an acquisition system, as previously described [14].

The concentration-relaxation response curves to acetylcholine (10^−9^–10^−4^ M) were analyzed in rings pre-contracted to the same tension with phenylephrine (0.3 × 10^−6^ and 10^−6^ M, in WKY-H and SHR, respectively). The concentration–relaxation response curves to nitroprusside (10^−10^–10^−6^ M) were obtained at dark in rings without endothelium precontracted to the same tension with phenylephrine.

Endothelium-dependent contractions to acetylcholine were tested in aortic rings initially stimulated with 80 mM KCl. After washing in the Krebs solution, and incubating for 30 min with N-nitro-L-arginine methyl ester (L-NAME) (10^−4^ M), increasing doses of acetylcholine were added (10^−9^–10^−4^ M). The contractile responses to acetylcholine were expressed as a percentage of the response to KCl.

### 2.7. Statistical Analysis

All variables data are presented as the mean values ± standard error of the mean (SEMs). The normality of variables was evaluated with the Kolmogorov and Shapiro-Wilk tests. In all cases, more than 95% of the data were analyzed. One-factor ANOVA or Kruskal-Wallis tests were used (depending on whether the normality assumption was met). To identify the difference between groups, the Bonferroni post hoc test was used. A *p* < 0.05 value was considered significant. The Statistical Package for the Social Sciences version 20 software was used to perform the statistical analysis (SPSS Inc., Chicago, IL, USA).

## 3. Results

### 3.1. Time-Course of Body Weight, Food Intake, Water Intake, and Diuresis

**Figure 1:**
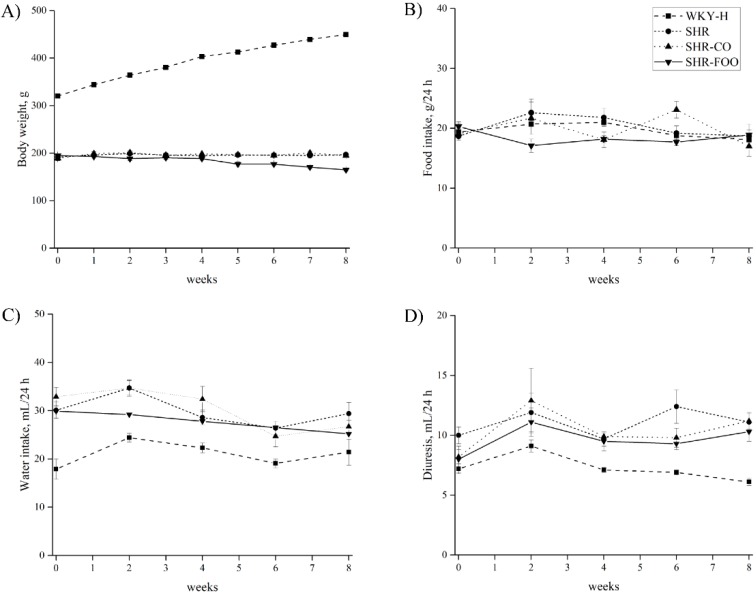
Time courses of BW (**A**), food intake (**B**), water intake (**C**), and diuresis (**D**) in the experimental groups. Data are means ± SEM. The ANOVA test was used to compare results among groups for normal distribution variables. The Bonferroni post hoc test was used for multiple comparisons among groups. *p* < 0.05 was considered significant. BW, body weight. SHR, spontaneously hypertensive rats. SHR-CO, spontaneously hypertensive rats supplemented with the control olive oil. SHR-FOO, spontaneously hypertensive rats supplemented with the functional olive oil. WKY-H, Wistar Kyoto healthy rats.

Body weight, food and water intake, and diuresis time-courses are shown in Figure 1. Body weight was lower in all SHR compared with the WKY-H rats, which is characteristic in these strains. No significant differences were found in treated and untreated SHR groups, which indicates that no toxic effects are associated with the enriched olive oil consumption, despite a slight decrease in body weight after the fourth week of the study. Food intake was around 20 g/day in all groups along the eight weeks of intervention, without significant differences among groups. Water intake was similar in the four experimental groups. Diuresis also increased in all SHR groups compared with the healthy WKY-H animals from the fourth week of treatment.

### 3.2. Effects of Enriched Olive Oil on Blood Pressure in SHR

**Figure 2:**
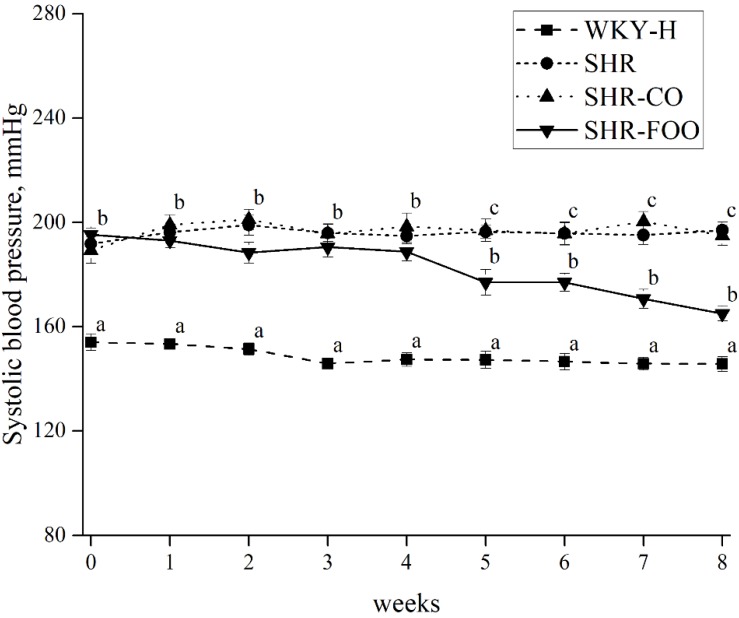
Time course of tail SBP measured by tail-cuff plethysmography in the experimental groups. Data are mean ± SEM. The ANOVA test was used to compare results among groups for normal distribution variables. The Bonferroni *post hoc* test was used for multiple comparisons among groups. *p* < 0.05 was considered significant. Different superscript letters indicate significant differences between post-intervention results (a,b,c). SHR, spontaneously hypertensive rats. SHR-CO, spontaneously hypertensive rats supplemented with the control olive oil. SHR-FOO, spontaneously hypertensive rats supplemented with the functional olive oil. WKY-H, Wistar Kyoto healthy rats.

Figure 2 depicts the time course of the tail SBP in the groups. Tail SBP was around 40 mmHg higher in SHR untreated group with respect to the WKY-H one throughout the study. Progressive reduction in SBP was detected at the end of the fourth week of FOO administration, which became significant from the fifth week (*p* = 0.006), and reached maximum at the eighth week of treatment (*p* = 0.001) versus the control SHR group (−30.3 ± 3.2 mmHg, with respect to the SHR untreated group). This decrease did not reach the values of control WKY-H, remaining 19.3 ± 0.3 mmHg higher than the healthy animals at that time. Supplementary Figure S1 shows SBP at the beginning and at the end of the intervention. A significant reduction of SBP is observed after the eight weeks of treatment with the FOO compared with the baseline (*p* = 0.001).

### 3.3. Vasoactive Peptides: Plasma Angiotensin II and Urinary Endothelin-1

**Figure 3:**
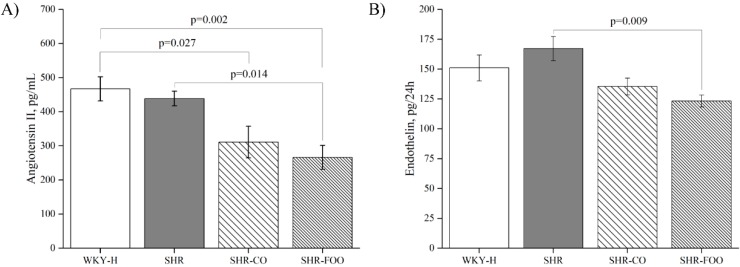
Vasoactive peptide concentrations after EIGHT weeks of intervention: plasma angiotensin II (**A**), urinary endothelin-1 (**B**), and data are mean ± SEM. The ANOVA test and the Bonferroni post hoc were used to compare results among groups. *p* < 0.05 was considered significant. SHR: spontaneously hypertensive rats. SHR-CO, spontaneously hypertensive rats supplemented with the control olive oil. SHR-FOO, spontaneously hypertensive rats supplemented with the functional olive oil. WKY-H, Wistar Kyoto healthy rats.

Sustained administration of the enriched EVOO produced a significant decrease in plasma levels of angiotensin II in SHR-FOO when compared with SHR. In addition, plasma angiotensin II levels tended to be lower after administration of the control olive oil compared with the SHR control group (*p* = 0.125). Differences were also found between the two supplemented groups of rats and the WKY-H animals (Figure 3A). Total urinary excretion of endothelin-1 showed a similar pattern to that observed for plasma angiotensin II. Thus, the SHR-FOO group had lower levels of urinary endothelin-1 compared with the SHR, while endothelin-1 levels tended to be lower after treatment with the control oil (*p* = 0.102) (Figure 3B).

### 3.4. Morphological Variables

At the end of the study period, the SHR groups had significantly higher heart and left ventricular weight in absolute values (HW and LVW, respectively) compared with the WKY-H group, as well as the indices relative to body weight (HW/BW and LVW/BW, respectively). These variables were significantly lower in SHR-FOO when compared with the SHR control group. The kidney weight in absolute values (KW) or the indices relatives to body weight (KW/BW) were similar in the four groups of rats (Table 2).

### 3.5. Plasma Urine Biochemical Variables

Table 3 shows plasma and urine biochemical variables. There were no significant differences in plasma sodium, urea, HDL cholesterol, and LDL cholesterol among groups. Total cholesterol was lower in the SHR-FOO than in the WKY-H group (*p* = 0.03), and tended to be lower compared with the SHR group (*p* = 0.106). Plasma creatinine was higher in the SHR control group when compared with the olive oil treated groups and the WKY-H animals.

Diuresis and urine creatinine excretion were higher in all hypertensive rats after eight weeks of treatment compared with healthy WKY. However, creatinine clearance was significantly lower after the treatment with both the CO and the FOO compared with the control SHR and WKY-H animals. Natriuresis was higher in the animals treated with the CO compared with the healthy WKY. Urinary nitrite levels were significantly higher in the FOO group compared with the SHR and WKY control groups (*p* = 0.004 and *p* = 0.04, respectively), and in the SHR-CO group compared to the SHR group (*p* = 0.028). No differences were observed between groups in kaliuresis, proteinuria, and water and sodium balances among groups (Table 3).

### 3.6. Oxidative and Inflammatory Biomarkers

Table 4 shows the urinary oxidative and plasma inflammatory biomarkers after eight weeks of treatment. Both urinary 8-OH-dG and F_2_-isoprostanes excretion was lower in all SHR animals when compared with healthy WKY. In addition, after the treatment with the FOO, 8-OH-dG was lower than in the SHR and SHR-CO groups. No effect of the olive oil treatment was observed with regard to urinary F_2_-isoprostanes levels when compared with the SHR group.

Plasma TNF-α was lower after the treatment with the FOO when compared with the healthy animals. No difference was observed between groups in plasma interleukin IL-6 and VEGF (Table 4).

### 3.7. Ex-Vivo Effects on Vascular Function 

**Figure 4:**
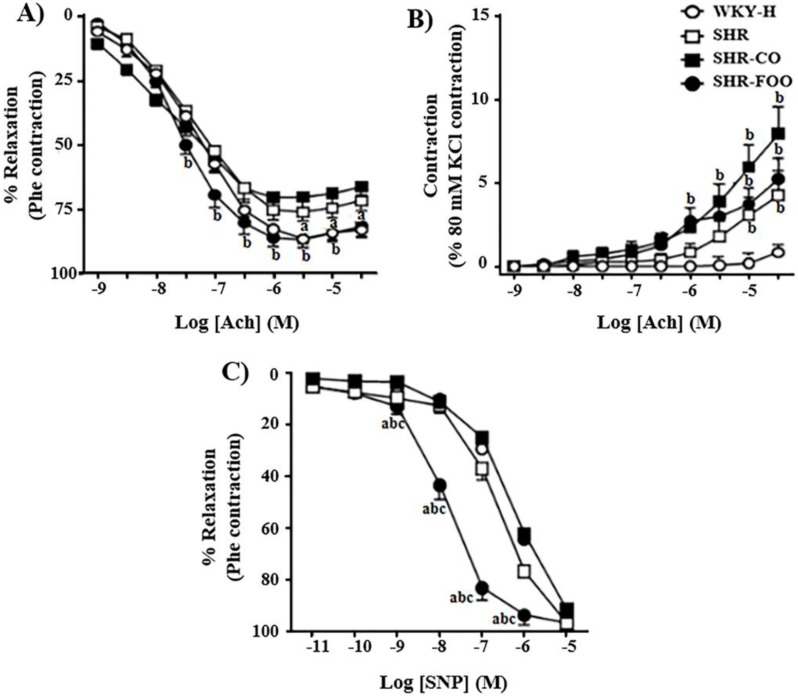
Ex vivo endothelium-dependent vasodilator responses to acetylcholine (**A**), endothelium-dependent vasoconstrictor responses to acetylcholine in the presence of L-NAME (10^−4^ M) (**B**), and endothelium-dependent vasodilator responses to nitroprusside (**C**) in aortic rings. Data are means ± SEM. ^a^ *p* < 0.05 compared with SHR control rats; ^b^ *p* < 0.05 compared with the WKY healthy group. ^c^ *p* < 0.05 compared with SHR-CO rats. SHR, spontaneously hypertensive rats. SHR-CO, spontaneously hypertensive rats supplemented with the control olive oil. SHR-FOO, spontaneously hypertensive rats supplemented with the functional olive oil. WKY-H, Wistar Kyoto healthy rats.

Aortae endothelium-dependent vasodilator responses to acetylcholine were higher in healthy WKY and in FOO-supplemented animals, at the higher concentrations in arteries stimulated with phenylephrine, when compared to aortae from control SHR (Figure 4A). In the presence of L-NAME (10^−4^ M), aortae from the three groups of SHR showed an increased endothelium-dependent vasoconstrictor response to acetylcholine than those obtained from WKY rats. Supplementation with the two olive oils did not significantly modify the endothelium-dependent vasoconstriction with respect to untreated SHR (Figure 4B). To analyze whether the changes in the responsiveness to acetylcholine are due to a fault in NO signaling in the vascular smooth muscle, we evaluated the effect of nitroprusside, which is a molecule that activates soluble guanylyl cyclase in vascular smooth muscle, mimicking the effects of endogenous NO. The concentration-response curves to the endothelium-independent vasodilator nitroprusside were similar in WKY, SHR, and SHR-CO animals, but were markedly increased in aortae from FOO-treated rats when compared with the rest of the experimental groups (Figure 4C).

## 4. Discussion

Olive oil is the main lipid source of the Mediterranean diet. Adhesion to this diet could contribute to the prevention of age-related hypertension [15]. The Mediterranean diet prevention study (PREDIMED) reported lower values of diastolic BP (DBP) in individuals consuming a Mediterranean diet with EVOO when compared with subjects consuming a low-fat one [16]. The main finding of the current study is that the daily treatment with 1 mL of an EVOO, enriched with bioactive compounds, obtained from the olive fruits and leaves (750 mg/kg), reduced SBP and vasoactive peptides: angiotensin II and endothelin-1, and increased urinary NO, after eight weeks of treatment in an experimental model of genetically hypertensive rats. The beneficial effect on these biomarkers was also found after the treatment with a control EVOO containing only 17.6 mg/kg of phenolic compounds, but not reflected in an SBP change. Our data also show additional health benefits, such as the improvement of cardiac hypertrophy, 8-OH-dG, and ex-vivo endothelial dysfunction in the group treated during eight weeks with the enriched EVOO when compared with the control hypertensive rats. 

The progressive anti-hypertensive effect of the enriched EVOO was observed from the fifth week of intervention, and was accompanied by a decreased cardiac hypertrophy (reflected in the lower left ventricular weight) after eight weeks of treatment. Sustained consumption, during at least two weeks of a diet enriched with EVOO, prevented the increase of SBP in younger SHR rats, without cardiac hypertrophy reduction [8]. In our study, the anti-hypertensive effect was not confirmed after treatment with CO. This indicates that the effect may be related to the presence of phenolic compounds in the EVOO. A previous study has reported that blood pressure decreased after two weeks of oral administration of an oleuropein-enriched olive leaf extract (15%), and, subsequently, cardiac hypertrophy also decreased after five weeks of its administration [12]. In addition, healthy subjects who consumed an oleuropein extract showed a decrease in SBP and DBP after six weeks of treatment [17]. These results support clinical data proposing that VOO is more efficient than any other type of dietary oil at reducing blood pressure. Moreover, this effect is not only mediated through the VOO MUFA content, but also through its bioactive minor components, mainly polyphenols [18,19]. However, the knowledge concerning the mechanisms underlying the antihypertensive effect of olive oil components is still lacking.

Endothelial dysfunction, which is a critical event in the development of hypertension, is controlled by several vasoactive peptides. Among them, angiotensin II and endothelin-1 actively contribute to the pathogenesis of hypertension and its complications [20]. The sustained presence of angiotensin II type 1 receptor antagonists prevents and attenuates the development of hypertension, cardiac hypertrophy, oxidative stress, and renal injury in SHR [21]. In addition, hypertension is associated with impaired NO production and bioavailability [22]. Our results show that VOO, independently of the polyphenol content, produced a decrease in plasma angiotensin II and urinary endothelin-1 concentrations, and, in parallel, an increase of NO. These effects may contribute to a decrease in the SBP after treatment with FOO, but their change was not enough to decrease SBP after the intake of the control oil, which indicates that other mechanisms might be implicated in the anti-hypertensive effect of the enriched oil. Previous authors have reported that olive oil intake induces changes in serum angiotensinase activity in animals [23] and humans [24]. In addition, the aqueous extract of *Olea europaea* leaves has been associated with the inhibition of ACE in vitro [25]. Storniolo et al. [26] demonstrated that hydroxytyrosol and a polyphenol-enriched EVOO extract reduced in vitro endothelin-1 secretion in experimental conditions. They simulated hyperglycemia and high free fatty acid levels observed in diabetes, which suggests that VOO polyphenols are involved in endothelial protection [26]. The present data are in accordance with previous reports concerning the fact that human consumption of olive oil rich in polyphenols decreases endothelin-1 both in vivo and ex vivo [27]. The lower angiotensin II plasma levels observed after the administration of the two EVOO may influence endothelin-1 modification. This could be a similar process since it occurs with angiotensin II inhibitors, such as losartan, which reduce urinary endothelin in the renal cortex of SHR [21], or enalapril, which decreases plasma endothelin levels in SHR [28]. Martin-Pelaez et al. [29] reported that supplementation with 25 mL/day of VOO containing 366 mg/kg of phenolic compounds modulates the expression of some of the genes related to the renin-angiotensin-aldosterone system, which proposed a possible mechanism underlying the reported decrease of SBP. In addition, it is possible that the endothelin-1 modification is involved in the increase of creatinine clearance produced by enriched EVOO, since several authors have suggested that kidney-derived endothelin exerts considerable renal effects, i.e.; decreasing the glomerular filtration rate and renal blood flow [30,31].

Other mechanisms may underlie differences observed between the two treatments. Enriched EVOO improved the ex-vivo acetylcholine-altered responses observed in aortae from SHR, which indicates a protective role in agonist-induced NO bioactivity. These results are in agreement with previous data showing that sustained oleuropein treatment ameliorated endothelium–dependent acetylcholine-induced vasodilation [12]. SHR showed an increased endothelium-dependent vasoconstriction induced by acetylcholine, which could be attributed to an increased endothelial release of vasoconstrictor prostanoids [32]. Acetylcholine-induced vasoconstriction was not affected by enriched EVOO, which suggests that the reduction in the release of endothelium dependent constrictor factors, such as TXB_2_ or TXA_2_, could not participate in the blood pressure lowering effect induced in this model. In the present study, the relaxing response to nitroprusside was augmented in the aortae from the enriched-oil treated group. This mechanism is not usually observed with antihypertensive agents, which suggests that an increased sensitivity to NO might improve endothelial dysfunction and blood pressure. Thus, and in accordance with the in vivo data, the differential functional changes observed in endothelium-dependent relaxation induced by the enriched EVOO could be attributed to the modulation of NO sensitivity.

Anti-hypertensive treatments are usually accompanied by a reduction in oxidative stress in SHR [12,21,28]. The antioxidant properties of olive oil polyphenols have been demonstrated in vitro [33], in experimental models [34], and in clinical trials [3,35]. A recent meta-analysis has suggested that a moderate polyphenol contain 56 mg polyphenols/L may be sufficient to maintain 8-OH-dG production [36]. Our results show that the urinary excretion of oxidative stress biomarkers was lower after supplementations with the enriched EVOO but not after the intake of the control VOO containing 17.6 mg/kg of phenolic compounds. These data concur with the reduced oxidative stress produced by an oleuropein-rich olive leaf extract in hypertensive/diabetic rats [37], and in rats fed a carbohydrate-high fat diet [38]. It has been suggested that different minor components of EVOO, such as hydroxytyrosol and/or oleuropein, probably mediated the antioxidant effect of EVOO [8,39]. In addition, the antioxidant effects of the enriched EVOO might be influenced by the anti-hypertensive effect, directly or mediated by the reduction of plasma levels of angiotensin II, since this molecule induces nicotinamide adenine dinucleotide phosphate oxidase (NADPH) activation [40,41], and its blockade reduces oxidative stress [21]. In accordance with previous studies [35], we did not find effects of VOO treatments on F2-isoprostanes as biomarkers of lipid oxidation. Anti-inflammatory effects of EVOO have been previously described [36,42]. A systematic review has concluded that the sustained consumption of VOO may reduce the plasma levels of IL-6 and TNF-α when associated with the Mediterranean diet and healthy lifestyle [43]. However, in the present work, we did not observe any effect of VOO treatments on plasma inflammatory biomarkers, probably because it is not associated to a Mediterranean pattern. 

We observed higher levels of oxidative and inflammatory biomarkers in healthy animals. Since the link between oxidative stress and a systemic inflammation is well described [44], our results are consistent since both oxidative and inflammatory biomarkers are lower in SHR than in WKR. To our best knowledge, no previous studies have compared oxidative and inflammatory biomarkers in healthy WKR versus this model of genetic hypertension. Thus, more studies are needed to clarify the antioxidant and anti-inflammatory effects of enriched-VOOs in other experimental animal models and in human trials.

Lastly, there is no consensus about the effect of VOO on plasma cholesterol levels. Our data show that total cholesterol was reduced in SHR after the enriched EVOO treatment, which indicates a possible hypo-cholesterolemic effect. This decrease is not associated with any particular lipoprotein. Tsartsou et al. [36] has revealed that a decrease of total cholesterol is related to the polyphenol contains [44], what is in accordance with the present results. 

## 5. Limitations and Strengths

Although the results obtained in animal model are promising, clinical trials are needed to confirm these results, particularly in pre-hypertensive and hypertensive humans. Assuming that the rats consume approximately 70 kcal/day and the average balanced diet in humans is 2000 kcal/day, the dose of olive oil administered in rats (1 mL/day) is comparable to an intake of olive oil in a human diet of 26 mL/day [45].

Knowledge on the molecular mechanisms responsible for benefits of VOO on hypertension could elucidate potential new pharmacological treatments and functional food, all of which would lead to better cardiovascular prevention.

## 6. Conclusions

In conclusion, these results demonstrate that sustained treatment with EVOO containing at least 17.6 mg/kg of phenolic compounds may decrease vasoconstrictor biomarkers and increase vasodilatory NO. In addition, enrichment with bioactive compounds (750 mg/kg of phenolic compounds, mainly hydroxytyrosol, 3,4-dihydroxyphenylglycol, and oleuropein) from the olive fruit and leaves may also lead to: (1) a reduction in hypertension and cardiac hypertrophy, (2) improved endothelial dysfunction, (3) a decreased oxidative status and (4) a reduced plasma total cholesterol levels in SHR. Therefore, the addition of an enriched EVOO to the diet may be a useful tool against high blood pressure and plasma cholesterol levels, which are two risk factors for cardiovascular diseases.

## Figures and Tables

**Table 1:** Characteristics of the olive oils used in the study.

Characteristics	CO	FOO
Acidity (%)	0.14	0.14
Peroxide value (meq/Kg)	5.3	9.5
K270	0.14	0.15
K232	1.79	1.85
DeltaK	<0.01	<0.01
**Main Fatty acids**		
Palmithic (C16) (%)	10.12	10.09
Stearic (C18) (%)	3.81	3.79
Oleic (C18:1) (%)	79.71	79.87
Linoleic (C18:2n6) (%)	3.95	3.91
Total ethyl esters (mg/Kg)	7	13
Total sterols (mg/Kg)	1316	1328
Total phenolic compounds (mg/Kg)	17.6	749.9

Individual phenolic compounds quantitative data obtained for the oils evaluated in this study by the LC-MS method described in Appendix A. Every result (expressed in mg/kg of olive oil) is the mean value of three independent replicates. RSD values were lower than 8.4% in every case. CO, control oil. FOO, functional oil. RSD, relative standard deviation

**Table 2:** Morphological variables in the experimental groups at the end of the experimental time.

	WKY-H	SHR	SHR-CO	SHR-FOO	*p*
BW(g)	444.9 ± 4.0 ^a^	403.4 ± 9.7 ^b^	394.9 ± 8.0 ^b^	390.5 ± 10.8 ^b^	0.001
HW (g)	1.0 ± 0.01 ^a^	1.3 ± 0.03 ^c^	1.3 ± 0.03 ^bc^	1.2 ± 0.03 ^b^	0.001
HW/BW (mg/g)	2.4 ± 0.02 ^a^	3.3 ± 0.1 ^c^	3.3 ± 0.08 ^bc^	3.1 ± 0.07 ^b^	0.001
LVW (mg)	0.86 ± 0.01 ^a^	1.1 ± 0.02 ^c^	1.2 ± 0.03 ^bc^	1.0 ± 0.03 ^b^	0.001
LVW/BW (mg/g)	1.9 ± 0.02 ^a^	2.8 ± 0.09 ^c^	3.0 ± 0.08 ^bc^	2.6 ± 0.05 ^b^	0.001
KW (g)	1.3 ± 0.02	1.2 ± 0.04	1.2 ± 0.03	1.2 ± 0.02	0.020
KW/BW (mg/g)	2.9 ± 0.05	3.1 ± 0.09	3.0 ± 0.06	3.1 ± 0.08	0.147

Data are means ± SEM. The ANOVA test was used to compare results among groups for normal distribution variables, and the Kruskal Wallis test for non-normal distribution variables. The Bonferroni *post hoc* test was used for multiple comparisons among groups. *p* < 0.05 was considered significant. Different superscript letters indicate significant differences between post-intervention results (a,b). BW, body weight. HW, heart weight. KW, kidney weight. LVW, left ventricular weight. SEM, standard error of the mean. SHR, spontaneously hypertensive rats. SHR-CO, spontaneously hypertensive rats supplemented with the control olive oil. SHR-FOO, spontaneously hypertensive rats supplemented with the functional olive oil. WKY-H, Wistar Kyoto healthy rats.

**Table 3:** Plasma and urine biochemical variables in the experimental groups after eight weeks of intervention.

	WKY-H	SHR	SHR-CO	SHR-FOO	*p*
**Plasma**					
Sodium (mEq/L)	144.1 ± 0.7	143.9 ± 1.2	144.7 ± 0.9	144.1 ± 0.8	0.940
Potassium (mEq/L)	4.3 ± 0.1 ^ab^	4.7 ± 0.2 ^b^	4.2 ± 0.1 ^a^	4.3 ± 0.1 ^ab^	0.040
Creatinine (mg/dL)	0.4 ± 0.03 ^a^	0.5 ± 0.05 ^b^	0.4 ± 0.03 ^a^	0.4 ± 0.03 ^a^	0.004
Urea (mg/dL)	37.5 ± 1.4	41.2 ± 2.8	41.9 ± 1.9	40.0 ± 1.4	0.400
Total cholesterol (mg/dL)	79.9 ± 3.3 ^a^	77.9 ± 5.9 ^ab^	74.3 ± 2.2 ^ab^	65.2 ± 1.9 ^b^	0.020
HDL cholesterol (mg/dL)	63.2 ± 2.6	63.6 ± 5.0	59.4 ± 2.0	63.2 ± 2.7	0.770
LDL cholesterol (mg/dL)	9.2 ± 0.7	10.8 ± 1.1	10.0 ± 0.7	9.2 ± 0.7	0.500
**Urine**					
Diuresis (mL/100 g/24 h)	1.3 ± 0.1 ^a^	2.7 ± 0.2 ^b^	2.8 ± 0.1 ^b^	2.7 ± 0.2 ^b^	0.001
Natriuresis (µEq/100 g/24 h)	243.7 ± 31.1 ^a^	285.8 ± 27.0 ^ab^	336.7 ± 20.9 ^b^	248.4 ± 18.1 ^ab^	0.040
Kaliuresis (µEq/100 g/24 h)	520.5 ± 50.8	609.4 ± 40.9	617.7 ± 37.9	531.5 ± 50.0	0.320
Creatinine (mL/min/kg)	1.8 ± 0.1 ^a^	2.7 ± 0.1 ^b^	2.8 ± 0.1 ^b^	2.5 ± 0.1 ^b^	0.001
Clearance/Creatinine (mL/min/kg)	0.64 ± 0.07 ^a^	0.63 ± 0.05 ^a^	0.94 ± 0.06 ^b^	0.91 ± 0.08 ^b^	0.001
Proteinuria (g/dL)	2.8 ± 2.3	2.9 ± 2.1	2.6 ± 1.0	2.3 ± 2.1	0.200
Nitrites µM	208.1 ± 43.9 ^ab^	144.8 ± 50.1 ^a^	344.4 ± 41.5 ^bc^	393.3 ± 48.7 ^c^	0.002
Water balance (mL/100 g/24 h)	3.4 ± 0.6	4.6 ± 0.7	3.9 ± 0.3	3.6 ± 0.2	0.510
Sodium balance (µEq/100 g/day)	462.9 ± 38.7	528.4 ± 45.7	415.4 ± 82.1	587.4 ± 61.7	0.210

Data are means ± SEM. The ANOVA test was used to compare results among groups for normal distribution variables, and the Kruskal Wallis test for non-normal distribution variables. The Bonferroni post hoc test was used for multiple comparisons among groups. *p* < 0.05 was considered significant. Different superscript letters indicate significant differences between post-intervention results (a,b). SEM, standard error of the mean. SHR, spontaneously hypertensive rats. SHR-CO, spontaneously hypertensive rats supplemented with the control olive oil. SHR-FOO, spontaneously hypertensive rats supplemented with the functional olive oil. WKY-H, Wistar Kyoto healthy rats.

**Table 4:** Oxidative stress and inflammatory biomarkers after eight weeks of intervention.

	WKY-H	SHR	SHR-CO	SHR-FOO	*p*
**Urine**					
8-OH-dG (ng/mL)	53.2 ± 5.0 ^a^	26.7 ± 4.2 ^b^	24.5 ± 4.4 ^b^	17.1 ± 4.4 ^c^	0.001
F_2_-isoprostanes (ng/mL)	7.6 ± 0.8 ^a^	3.8 ± 0.5 ^b^	2.8 ± 0.3 ^b^	3.3 ± 0.3 ^b^	0.001
**Plasma**					
IL-6 (pg/mL)	3.6 ± 0.1	3.3 ± 0.1	3.4 ± 0.1	3.3 ± 0.2	0.280
TNF-α (pg/mL)	45.9 ± 8.9 ^b^	26.3 ± 2.7 ^ab^	24.4 ± 3.2 ^ab^	23.6 ± 3.2 ^a^	0.021
VEGF (pg/mL)	12.9 ± 1.3	10.6 ± 0.7	9.6 ± 0.5	11.5 ± 0.7	0.060

Data are means ± SEM. The ANOVA test was used to compare results among groups for normal distribution variables, and the Kruskal Wallis test for non-normal distribution variables. The Bonferroni post hoc test was used for multiple comparisons among groups. *p* < 0.05 was considered significant. Different superscript letters indicate significant differences between post-intervention results (a,b). IL-6, interleukin 6. SEM, standard error of the mean. SHR, spontaneously hypertensive rats. SHR-CO, spontaneously hypertensive rats supplemented with the control olive oil. SHR-FOO, spontaneously hypertensive rats supplemented with the functional olive oil. TNF-α, tumor necrosis factor alpha. VEGF, vascular endothelial growth factor. WKY-H, Wistar Kyoto healthy rats.

## Supplementary Materials

The following are available online at https://www.mdpi.com/2072-6643/11/8/1728/s1, Table S1: Detailed composition of the olive oils used in the study. Figure S1: Systolic blood pressure at the beginning and end of the intervention. The data are means ± SEM. The ANOVA test were used to compare results between groups for a normal distribution variable, and for multiple comparisons among groups. The Bonferroni post hoc test was used. *p* < 0.05 was considered significant. SHR, spontaneously hypertensive rats. SHR-CO, spontaneously hypertensive rats supplemented with the control olive oil. SHR-FOO, spontaneously hypertensive rats supplemented with the functional olive oil. WKY-H, Wistar Kyoto healthy rats.

## Appendix A

Quantitative determination of phenolic compounds in the used EVOOs.

The quantitative characterization of the oils that were administered to the rats was carried out by applying a slightly modified liquid chromatography coupled to mass spectrometry (LC-MS) multi-class methodology previously reported elsewhere [13]. In short, 1 (± 0.01) g of EVOO was subjected to a three steps liquid-liquid extraction protocol with ethanol/water mixtures in different proportions. Then, the supernatants were collected together, evaporated to dryness and re-dissolved in 1 mL of ethanol/water (80:20, v/v). After filtering them through a 0.22-μm nylon syringe filter, the prepared extracts were injected into the LC-MS system consisting of an Agilent 1260 LC (Agilent Technologies, Waldbronn, Germany) coupled to a Bruker Daltonics Esquire 2000TM ion trap MS (Bruker Daltonik, Bremen, Germany) by means of an electrospray interface. Chromatographic separation was carried out at 40 °C in a Zorbax Extend C18 column (4.6 x 100 mm, 1.8 μm particle size) (Agilent Technologies) by applying a 30-minute mobile phase gradient program using acidified water and acetonitrile at a flow rate of 1 mL/min. Full scan MS spectra (50-1000 m/z) were acquired in negative polarity with a capillary voltage of + 3200 V from the beginning to 17 minutes and + 3500 V from that time to the end of the run. Source parameters were set as follows: 30 psi of nebulizer pressure, 9 L/min of drying gas flow, and 300 °C of drying gas temperature.

Compounds lacking commercially available standards were quantified in terms of a compound presenting a related chemical structure. Hydroxytyrosol calibration curve was used to quantify 3,4-dihydroxyphenylglycol and chlorogenic acid. Secoiridoids (in glycosylated and aglycone forms) were quantified in terms of oleuropein. Flavonoid glucoside isomers were quantified considering the corresponding 7-O-isomer. Luteolin was used for diosmetin quantification and syringaresinol and acetoxypinoresinol concentrations were calculated by comparison with the pinoresinol MS response.
