# Supplementary material for: Cardioprotective Effect of a Virgin Olive Oil Enriched with Bioactive Compounds in Spontaneously Hypertensive Rats
Source: Nutrients. 2019 Jul 26;11(8):1728. doi: 10.3390/nu11081728 (PMC6722946; doi:10.3390/nu11081728)
Supplement: Supplementary file 1 [file nutrients-11-01728-s001.pdf]

Supplemental Table S1. Characteristics of the olive oils used in the study.

	CO	FOO
Acidity (%)	0.14	0.14
Peroxide value (meq/Kg)	5.3	9.5
K270	0.14	0.15
K232	1.79	1.85
DeltaK	<0.01	<0.01
**Fatty acids**		
Miristic acid (C14) (%)	0.01	0.01
Palmitic acid (C16) (%)	10.12	10.09
Palmitoleic acid (C16:1) (%)	0.93	0.92
Margaric acid (C17) (%)	<0.10	<0.10
Margaroleic acid (C17:1) (%)	0.16	0.15
Stearic acid (C18) (%)	3.81	3.79
Oleic acid (C18:1) (%)	79.71	79.87
Linoleic acid (C18:2n6) (%)	3.95	3.91
Linolenic acid (C18:3n3) (%)	0.67	0.65
Araquidic acid (C20) (%)	0.33	0.32
Gadoleic acid (C20:1) (%)	0.19	0.18
Behenic acid (C22) (%)	0.11	0.11
Lignoceric acid (C24) (%)	<0.10	<0.10
**Total ethyl esters (mg/Kg)**	**6**	**13**
Ethyl palmitate (mg/Kg)	3	6
Ethyl oleate (mg/Kg)	4	7
**Total sterols (mg/Kg)**	**1316**	**1328**
Cholesterol (% of total sterols)	0.1	0.1
Brassterol (% of total sterols)	<0.1	<0.1
Campesterol (% of total sterols)	3.2	3.3
Stigmasterol (% of total sterols)	0.5	0.5
B-Sitosterol (% of total sterols)	94.9	94.8
D7-Stigmastenol (% of total sterols)	0.4	0.4
**Total phenolic compounds (mg/Kg)**	**17.6**	**749.9**
Organic and phenolic acids and aldehydes (mg/kg)	*n.d.*	*235.2*
Quinic acid* (mg/kg)	n.d.	92.3
3,4-dihydroxyphenylglycol* (mg/kg)	n.d.	116.6
Chlorogenic acid (mg/kg)	n.d.	22.2
p-coumaric acid* (mg/kg)	n.d.	3.0
Vanillin* (mg/kg)	n.d.	0.32
Ferulic acid* (mg/kg)	n.d.	0.80
Simple phenols (mg/kg)	*0.15*	*179.0*
Hydroxytyrosol* (mg/kg)	n.d.	110.0
Tyrosol* (mg/kg)	0.15	69.0
Glycosylated secoiridoids and derivatives (mg/kg)	*n.d.*	*204.8*
Acyclodihydroelenolic acid hexoside (mg/kg)	n.d.	21.0
Oleoside/secologanoside (mg/kg)	n.d.	103.2
Elenolic acid glucoside (mg/kg)	n.d.	31.9
Hydroxy oleuropein (mg/kg)	n.d.	32.3
Oleuropein* (mg/kg)	n.d.	16.4
Secoiridoids and derivatives in aglycone form (mg/kg)	*17.0*	*82.9*
Elenolic acid (mg/kg)	0.01	2.6

Hydroxydecarboxymethyl oleuropein aglycone (mg/kg)	n.d.	4.7
Decarboxymethyl oleuropein aglycone (mg/kg)	1.3	8.2
10-Hydroxy-oleuropein aglycone (mg/kg)	0.11	0.3
Decarboxymethyl ligstroside aglycone (mg/kg)	2.4	6.3
Ligstroside algycone (mg/kg)	6.1	2.2
Oleuropein algycone (mg/kg)	7.1	58.5
Flavonoids (mg/kg)	*0.1*	*40.9*
Luteolin-7-O-glucoside* (mg/kg)	n.d.	4.6
Luteolin-O-glucoside isomers (mg/kg)	n.d.	12.3
Apigenin-7-O-glucoside* (mg/kg)	n.d.	1.7
Apigenin-O-glucoside isomers (mg/kg)	n.d.	3.6
Luteolin* (mg/kg)	0.03	14.5
Apigenin* (mg/kg)	0.04	1.6
Diosmetin (mg/kg)	0.03	2.5
Lignans (mg/kg)	*0.31*	*7.1*
Syringaresinol (mg/kg)	0.10	1.8
Pinoresinol* (mg/kg)	0.11	2.5
Acetoxypinoresinol (mg/kg)	0.10	2.8

Individual phenolic compounds quantitative data obtained for the oils evaluated in this study by the LC-MS method described in Appendix A. Every result (expressed in mg/kg of olive oil) is the mean value of three independent replicates. Individual sterols are expressed as the percentage of total sterols. RSD values were lower than 8.4 % in every case. *Phenolic compounds quantified in terms of their pure standard. CO, control oil FOO, functional oil; n.d.: non detected.

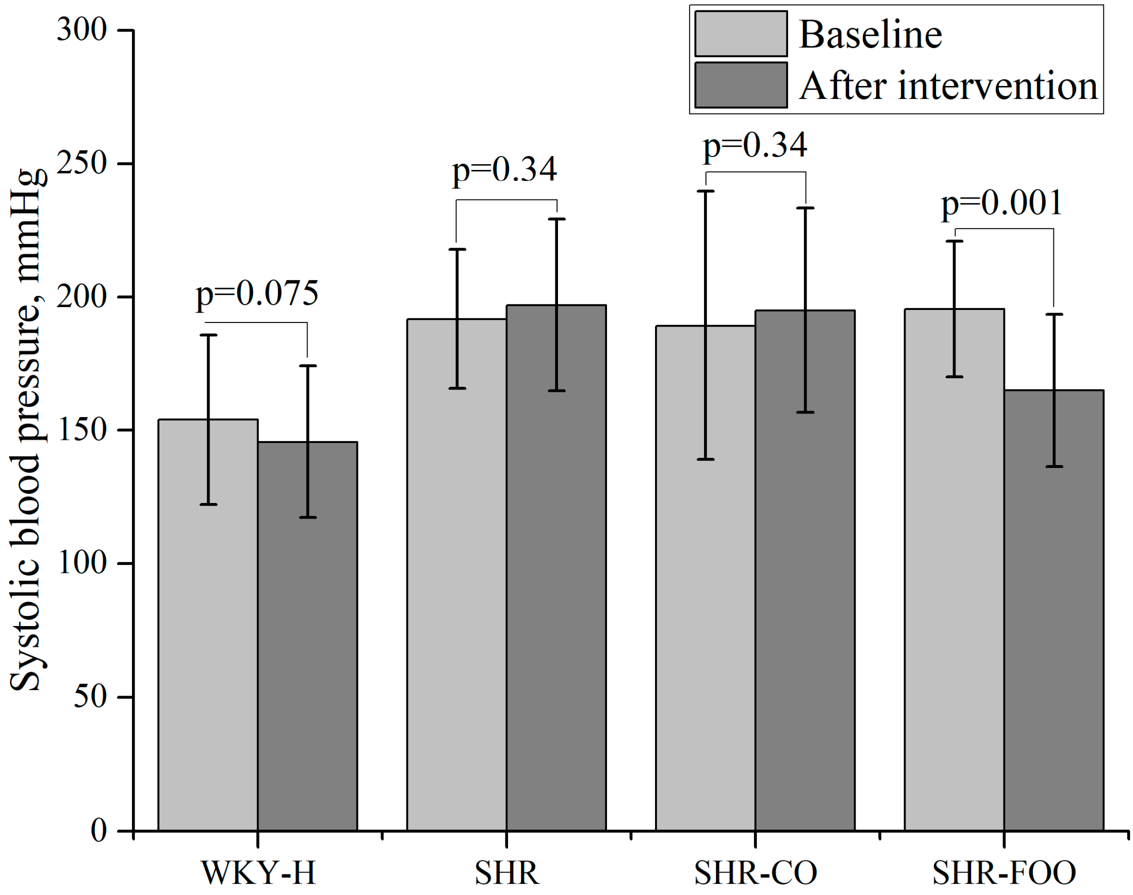

Supplemental Figure S1: Systolic blood pressure at the beginning and end of the intervention. The data are means ± SEM. ANOVA test were used to compare results between groups for normal distribution variable, for multiple comparisons among groups Bonferroni *post hoc* test was used. $P < 0.05$ was considered significant. SHR, spontaneously hypertensive rats. SHR-CO, spontaneously hypertensive rats supplemented with the control olive oil. SHR-FOO, spontaneously hypertensive rats supplemented with the functional olive oil. WKY-H, Wistar Kyoto healthy rats.
